# Devil's Claw to Suppress Appetite—Ghrelin Receptor Modulation Potential of a *Harpagophytum procumbens* Root Extract

**DOI:** 10.1371/journal.pone.0103118

**Published:** 2014-07-28

**Authors:** Cristina Torres-Fuentes, Wessel F. Theeuwes, Michael K. McMullen, Anna K. McMullen, Timothy G. Dinan, John F. Cryan, Harriët Schellekens

**Affiliations:** 1 Alimentary Pharmabiotic Centre, University College Cork, Cork, Ireland; 2 Department of Anatomy and Neuroscience, University College Cork, Cork, Ireland; 3 Life Force Research, Ljungskile, Sweden; 4 School of Biosciences, University of Westminster, London, United Kingdom; 5 Dept of Psychiatry, University College Cork, Cork, Ireland; Monash University, Australia

## Abstract

Ghrelin is a stomach-derived peptide that has been identified as the only circulating hunger hormone that exerts a potent orexigenic effect via activation of its receptor, the growth hormone secretagogue receptor (GHS-R1a). Hence, the ghrelinergic system represents a promising target to treat obesity and obesity-related diseases. In this study we analysed the GHS-R1a receptor activating potential of *Harpagophytum procumbens*, popularly known as Devil's Claw, and its effect on food intake *in vivo*. *H. procumbens* is an important traditional medicinal plant from Southern Africa with potent anti-inflammatory and analgesic effects. This plant has been also used as an appetite modulator but most evidences are anecdotal and to our knowledge, no clear scientific studies relating to appetite modulation have been done to this date. The ghrelin receptor activation potential of an extract derived from the dried tuberous roots of *H. procumbens* was analysed by calcium mobilization and receptor internalization assays in human embryonic kidney cells (Hek) stably expressing the GHS-R1a receptor. Food intake was investigated in male C57BL/6 mice following intraperitoneal administration of *H. procumbens* root extract in *ad libitum* and food restricted conditions. Exposure to *H. procumbens* extract demonstrated a significant increased cellular calcium influx but did not induce subsequent GHS-R1a receptor internalization, which is a characteristic for full receptor activation. A significant anorexigenic effect was observed in male C57BL/6 mice following peripheral administration of *H. procumbens* extract. We conclude that *H. procumbens* root extract is a potential novel source for potent anti-obesity bioactives. These results reinforce the promising potential of natural bioactives to be developed into functional foods with weight-loss and weight maintenance benefits.

## Introduction

Ghrelin is a 28 amino acid peptide, which is synthetized mainly in the stomach, identified as the first and only known peripheral hormone exerting an appetite-stimulating effect through activation of its receptor, the growth hormone secretagogue receptor (GHS-R1a) [1]. Indeed, several studies have shown the orexigenic effects of ghrelin following central or peripheral administration [2]–[4]. Although this receptor is also implicated in the central secretion of the growth hormone (GH) from the anterior pituitary cells [5], its role in the appetite modulation is via activation of orexigenic neurons in the hypothalamic arcuate nucleus (ARC) (for review see [6].

A dysregulated appetite signalling, including aberrant ghrelin signalling, may contribute to the development of metabolic disorders such as obesity [7]. The World Health Organization (WHO) defines obesity and overweight as “abnormal or excessive fat accumulation that may impair health” [8]. The prevalence of obesity has been continuously increasing since 1980 and is reaching epidemic proportions in both developed and developing countries. Recent numbers show that more than 1.6 billion adults are overweight (BMI ≥25 kg/m^2^) and 400 million of them are clinically obese (BMI ≥30 kg/m^2^) [8]. In addition, obesity and being overweight are the fifth leading risk for mortality. The high rate of obesity has led to increases in complications associated with obesity, notably the Metabolic Syndrome (Reaven's Syndrome X; the Insulin Resistance Syndrome) which includes cardiovascular risk factors such as insulin resistance (type 2 diabetes), glucose intolerance, dyslipidemia and hypertension [8].

Moreover, current available anti-obesity therapeutics are limited and associated with adverse side effects, emphasising the urgent need for novel strategies contributing to the maintenance of a healthy weight [9], [10]. The ghrelinergic system represents a promising pharmacologic target for the treatment of obesity and obesity-related diseases. Indeed, several studies have shown that inhibition of the ghrelin signalling pathway results in a reduction of food intake through decreased appetite and increased energy expenditure and fat catabolism, underlining the potential of ghrelin receptor antagonists, inverse agonist and other strategies targeting the ghrelin hormone in the development of anti-obesity therapeutics (for review see[7], [11]–[15]).

Over the past decade, scientific research has demonstrated anti-obesity effects in bioactives from plants (for review see [16], [17]). Appetite modulating bioactives from natural resources may be an interesting alternative for current anti-obesity drugs contributing to an enhanced safety profile to treat metabolic disorders [18]. In this study, we investigate the effect of a plant extract derived from the dried tuberous roots of *Harpagophytum procumbens* on GHS-R1a receptor modulation *in vitro* and on food intake *in vivo*. This plant, commonly known as Devil's Claw, is a perennial herb from the Kalahari region of Southern Africa, where it has historically been used in traditional medicine with anti-inflammatory, analgesic, anti-oxidant, anti-diabetic, antimicrobial, anti-malarial, anticancer, hypotensive and cardiodepressant, anticonvulsant and uterotonic activities as well as an appetite modulator (for review see [19]–[21]). Therefore, this plant is becoming of interest and several animal, clinical and *in vitro* studies have investigated some of these properties, specially its anti-inflammatory and analgesic effects (for review see [21], [22]). Hence, effective treatments of inflammation, rheumatoid arthritis, tendonitis, osteoarthritis and dyspepsia have been shown (for review see [20]). However, scientific studies are lacking regarding the *H. procumbens* appetite modulation effect.

This study aims to investigate the ability of *H. procumbens* to control appetite via modulation of the GHS-R1a receptor. To this end, GHS-R1a receptor-mediated calcium influx is analysed using an *in vitro* calcium mobilization assay. Moreover, *H. procumbens* mediated anorexigenic effects are investigated *in vivo*.

## Materials and Methods

### Ethics Statement

All animal experiments were conducted in accordance with the European Directive 86/609/EEC, the Recommendation 2007/526/65/EC and approved by the Animal Experimentation Ethics Committee of University College Cork (Animal ethical permit number #2010/028). All efforts were made to minimise animal suffering and to reduce the number of animals used.

### Compositional analysis of *Harpagophytum procumbens*

The source of the material was a certified sample powder of unprocessed dried *Harpagophytum procumbens* root obtained from Proline Botanicals, Hull, UK (now trading as Herbs in a Bottle, Lincolnshire, UK). The dried *H. procumbens* root powder is green/brown in colour, not-irradiated and not derived from genetically modified material, as certified by the supplier.

In addition, the chemical composition of the dried *H. procumbens* root powder was analysed following different analytical methods. Ash and moisture were determined using association of analytical communities (AOAC) international methods 942.05, and 934.01 approved methods [23], respectively. Lipids were analysed according to the reference method 659:2009 from the International Organization for Standardization (ISO) [24]. Saccharides were determined according to Dubois *et al*., [25]. Total fibre content was determined according to Lee, Prosky and Devries [26]. Protein content was determined by amino acid analysis according to Hidalgo, Alaiz and Zamora [27]. Polyphenol content was determined using the Folin-Ciocalteou reagent as described by Singleton, Orthofer and Lamuela-Raventos [28] using a standard curve of catechin. Specific details on these methods are given in Text S1. The rest of the components (62.4%) were calculated by difference and might correspond to carbohydrates other than saccharides, such as insoluble carbohydrates [29].

### Cell culture

Human embryonic kidney cells (Hek293a) (Invitrogen, Dun Laoghaire, Ireland) were maintained in culture in high glucose Dulbecco's modified Eagle's medium (DMEM, Invitrogen) containing 10% heat inactivated fetal bovine serum (FBS) (Sigma-Aldrich, Wicklow, Ireland) and 1% non-essential amino acids (NEAA) (Gibco, Life Technologies, Dublin, Ireland) at culture conditions (37°C and 5% CO_2_ in a humidified atmosphere). Hek293a cells were transfected with a plasmid construct expressing the human GHS-R1a receptor as previously described [30] and cultured in complete DMEM media, containing 300 ng/µl G418 (Calbiochem, Merck KGaA, Darmstadt, Germany) as maintenance antibiotic. Cells were grown to a confluence of >85% and subsequently split to a lower density for continued culturing.

### Resazurin assay

Cytotoxicity of *H. procumbens* was determined using the resazurin assay (R&D systems, Inc.) according manufacturer's instructions. Resazurin is a blue non-toxic, water soluble, redox-sensitive dye that undergoes a colour change following reduction by viable cells. Absorbance of the colour change is measured at 570 nm. Hek293a cells were seeded in a 96-well microtiter plate at 2.8*10^5^ cells/ml (2.8*10^4^ cells/well) and maintained for 48 h at culture conditions. For the last 24 h of this time period, media was replaced with serum free DMEM media containing 1% NEAA. The dried *H. procumbens* root powder was dissolved in saline at 50 mg/mL containing 2.5% DMSO (Sigma-Aldrich). Then, it was centrifuged for 5 min at 2000 rpm and the supernatant was used to analyze its cytotoxicity. Cells were exposed for 4 h to this *H. procumbens* root extract at different concentrations up to 10 mg/mL, all comprising 10% resazurin dye. Cell viability was calculated as percentage of control (cells in 1x Hanks balanced salt solution, HBSS) (Gibco), supplemented with 20 mM HEPES (Sigma-Aldrich). Values above 90% are not considered cytotoxic.

### Calcium mobilization assay

**Figure 1:**
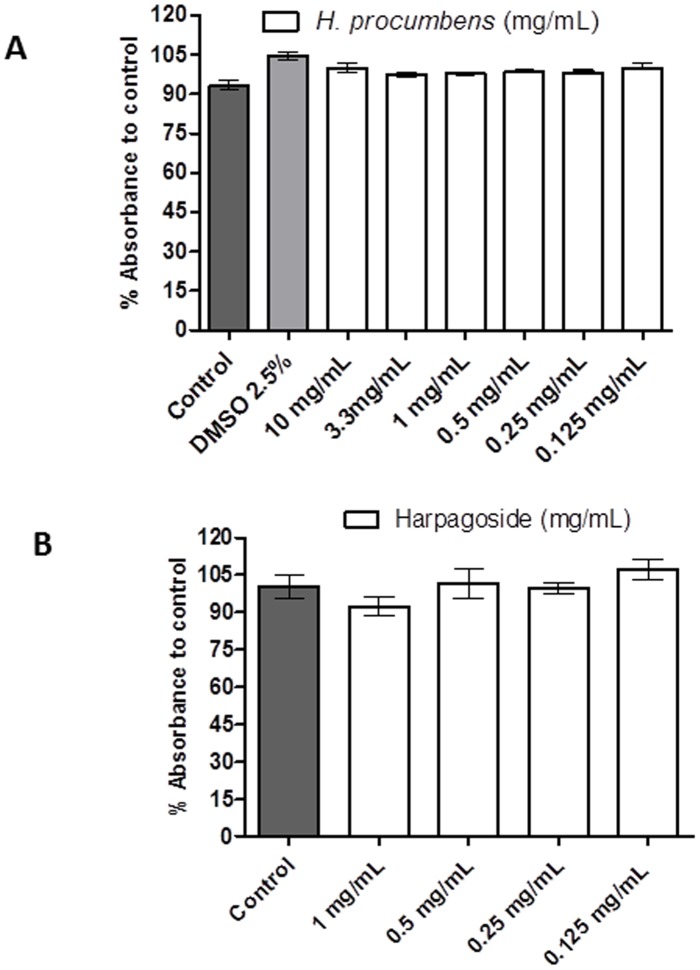
*H. procumbens* root extract has no cytotoxicity on Hek cells (Hek293a). Cellular viability was around 100% after exposure to different concentrations of *H. procumbens* root extract up to 10 mg/mL (A) or purified Harpagoside up to 1 mg/mL (B). Results are expressed as percentage of viability with respect to the control (cells in 1x HBSS containing 20 mM HEPES). Graph represents the mean ± SEM of triplicate samples from one representative assay.

G-protein coupled receptor-mediated changes in intracellular calcium (Ca^2+^) were determined using a Flex station II multiplate fluorometer (Molecular Devices Corporation, Sunnyvale, CA, USA). Calcium mobilization assays were performed based on protocols described in previous studies [31], [32]. Briefly, stably transfected Hek293a cells were seeded in black 96-well microtiter plates at a density of 2.8*10^5^ cells/ml (2.8*10^4^ cells/well) and maintained for ∼24 h at culture conditions. Next, growth media was replaced by serum free DMEM media containing 1% NEAA and the cells were incubated for a further ∼24 h at culture conditions. After removal of the serum free DMEM media, cells were incubated for 90 min with 25 µl of assay buffer (1x Hanks balanced salt solution, HBSS, containing 20 mM HEPES) and 25 µl of 1x Ca4 dye (Molecular Devices Corporation, Sunnyvale, CA, USA), according to the manufacturer's instructions. The dried *H. procumbens* root powder was dissolved in assay buffer at 100 mg/mL containing 10% DMSO. Then, the solution was centrifuged for 5 minutes at 2000 rpm and the supernatant was used for the assay. While we determined that 2.5% DMSO is not toxic to cells (**Figure 1**), the DMSO concentration exposed directly on the cells was no higher than 0.33% in this assay. Fluorescent readings were taken for a total of 80 seconds at 37°C in flex mode with excitation wavelength of 485 nm and emission wavelength of 525 nm. Addition of ghrelin or a serial dilution of the test compound, *H. procumbens* root extract, (25 µl/well) was performed by the Flexstation II after 16 secs during continuous fluorescent measurements for a total of 80 secs. The relative increase in intracellular calcium [Ca^2+^] was calculated as the difference between maximum and baseline fluorescence (Vmax-Vmin) and depicted as percentage relative fluorescent units (RFU) normalized to maximum response (100% signal) obtained with 3.3% FBS. Background fluorescence was obtained by cells in assay buffer alone and subtracted from RFUs. Exposure to the endogenous agonist ghrelin (Tocris, R&D Systems, Abingdon, UK), inverse agonist peptide [D-Arg1, D-Phe5, D-Trp7,9, Leu11]-substance P (SP) (Tocris, R&D Systems) and harpagoside (Santa Cruz Biotechnology, Inc.) were also carried out. Ghrelin and [D-Arg1, D-Phe5, D-Trp7,9, Leu11]-substance P were prepared in assay buffer. Exposure to *H. procumbens* extract following pretreatment to the inverse agonist [D-Arg1, D-Phe5, D-Trp7,9, Leu11]-substance P was also carried out. This pretreatment was performed during the calcium dye incubation. Harpagoside, which is the main iridoid glycoside in *H. procumbens*, was solubilised in methanol and further diluted in assay buffer containing a final concentration of 3% methanol. In the calcium assay, this translates to a maximum concentration of 1% methanol exposure to the cells. To directly compare the potency of the *H. procumbens* root extract with harpagoside, the dried root powder was also solubilised in methanol and further diluted in assay buffer to contain a 3% final methanol concentration. In a separate experiment no significant differences in potency were observed between the *H. procumbens* root powder solubilised in DMSO versus methanol (data not shown). No toxicity was observed in the resazurin assay for cells exposed either to up to 5% methanol or *H. procumbens* root powder dissolved in assay buffer (data not shown). Data was analysed using GraphPad Prism software (PRISM 5.0; GraphPAD Software Inc., San Diego, CA, USA). Sigmoidal dose-response curves were constructed using nonlinear regression analysis with variable slope, excluding values resulting from obvious incorrect pipetting by the Flexstation II.

### Internalization assay

Hek-GHSR1a-EGFP cells were seeded in a poly-L-lysine (Sigma-Aldrich) coated 96-well microtiter plate at 3*10^4^ cells per well and incubated for 48 h at culture conditions. For the last 24 h of this time period media was replaced with serum free DMEM media. Cells were treated with *H. procumbens* extract at 10 mg/mL containing 1% DMSO, prepared as previously described in the calcium mobilization assay, for 1 h at 37°C. The cells were fixed with 4% paraformaldehyde in phosphate buffer saline (PBS) for 20 min, washed once with PBS and stained with 5 µg/mL bisbenzimide (Sigma-Aldrich) for 5 min. After bisbenzamide staining, cells were washed three times with PBS and imaged on the GE Healthcare IN Cell Analyser 1000 (GE Healthcare, Buckinghamshire, UK) in PBS. Ghrelin was used as positive control. In addition, treatment with the inverse agonist, [D-Arg1, D-Phe5, D-Trp7,9, Leu11]-substance P, and the antagonist D[Lys3]-GHSR6 (Tocris, R&D Systems) were also carried out. The potential of compounds to internalize the receptor expressed with a C-terminal enhanced green fluorescent protein (EGFP) tag was analysed using ImageJ 1.46r software (National Institutes of Health, MD, USA). In total 12 individual cells across 3 independent images were analysed and fluorescence intensity of perinuclear receptor expression versus plasma membrane was determined. The single highest intracellular pixel intensity was compared to highest membrane pixel intensity along a straight line axis in each selected cell. The average pixel intensity ratio of each treatment was expressed as the mean ± SEM. Data were analysed and depicted using GraphPad Prism software (PRISM 5.0; GraphPAD Software Inc.).

### Cumulative food intake

Male C57BL/6 mice (purchased from Harlan laboratories, Derby, UK) were single-housed in standard holding cages. On date of arrival mice were 6–7 weeks of age. The holding room temperature (21±1°C) and humidity (55±10%) were controlled and under a 12 h light/dark cycle (lights on 7.00 AM, lights off 7.00 PM). Water and food (2018S Teklad Global 18% Protein Rodent Diet, Harlan laboratories) were *ad libitum* available during whole study unless indicated otherwise. The mice were habituated on three independent days to the experimental settings. Cumulative food intake studies, with ten animals per group, were performed based on protocols described in previous studies [33], [34]. The sample size is based on a power calculation aimed at detecting differences at the 0.05 level. Briefly, the body weight of the mice was determined and the mice were single-housed in new cages in the experimental room and habituated for 20 minutes before injections. *H. procumbens* root extract (500 mg/kg and 200 mg/kg in saline containing 2.5% DMSO) and vehicle (saline containing 2.5% DMSO) were administered via intraperitoneal (IP) administration (10 µl/gram of body weight). A pre-weighed chow food pellet was placed in the experimental cages 20 minutes after IP injection. Thereafter, the amount of food consumed was weighed in regular time intervals (20 min, 40 min, 1 h, 1 h30 min, 2 h, 3 h, 4 h, 5 h and 6 h). At the end of the experiment the mice were placed back in their original cages in the holding room. Data was analysed using GraphPad Prism software (PRISM 5.0; GraphPAD Software Inc.).

### Statistical analysis

Statistical analyses were performed using SPSS software (IBM SPSS statistics 20). Statistical analysis for calcium mobilization assay was performed using a Levene's Test for the analysis of the equality of the variances followed by an independent sample T-test, for internalization assay a one-way ANOVA with LSD post hoc test was used. Statistical analyses for *in vivo* studies were performed using a general linear model repeated measurement. Statistical significances are subsequently depicted as follows: * indicating *p*<0.05, ** indicating *p*<0.01 or *** indicating *p*<0.001.

## Results

### Chemical characterization of the dried *Harpagophytum procumbens* root powder

To allow a better understanding of the possible bioactives found in the dried *H. procumbens* root powder we analysed its chemical composition (**Table 1**). The dried *H. procumbens* root powder was poor in protein (1%), lipids (0.79%), polyphenols (1.16%) and saccharides (2.53%). The most abundant components were fibre (22.9%) and carbohydrates other than saccharides (62.4%). In addition, previous studies have identified iridoid glycosides as the main phytochemicals in *H. procumbens* (for review see [20]). These compounds are cyclopentanoid monoterpene-derived compounds with a glycoside bound as an O-linked glucoside and may, therefore, be present in this major fraction. One of the major iridoid glycosides described in *H. procumbens is* harpagoside [35] and has been included in this study.

**Table 1:** Chemical composition of the dried *H. procumbens* root powder.

Components	Dried *H. procumbens* root (g/100 g)
**Protein**	1.02±0.01
**Moisture**	5.76±0.06
**Ash**	3.41±0.06
**Lipids**	0.79±0.00
**Fibre**	22.91±0.14
**Polyphenols**	1.16±0.00
**Soluble carbohydrates**	2.53±0.06
**Insoluble carbohydrates** *	62.42

*calculated by difference.

### *Harpagophytum procumbens* root extract potently activates the GHS-R1a receptor *in vitro*

The potential cytotoxicity of *H. procumbens* root extract was analysed to test its suitability for cell culture studies. This was assessed by the resazurin assay, which is a widely used method to analyse viability of bacteria and mammalian cells [36]. Viability of Hek cells is depicted (**Figure 1**), calculated as percentage of control (cells in HBSS). Cells were exposed for 4 h to *H. procumbens* root extract at different concentrations, up to 10 mg/mL (**Figure 1A**) and purified Harpagoside (**Figure 1B**). No cytotoxic effects were observed (<90%), showing a cellular viability around 100% with respect to the control, which makes *H. procumbens* root extract a safe, suitable compound for the cellular calcium mobilization assay.

**Figure 2:**
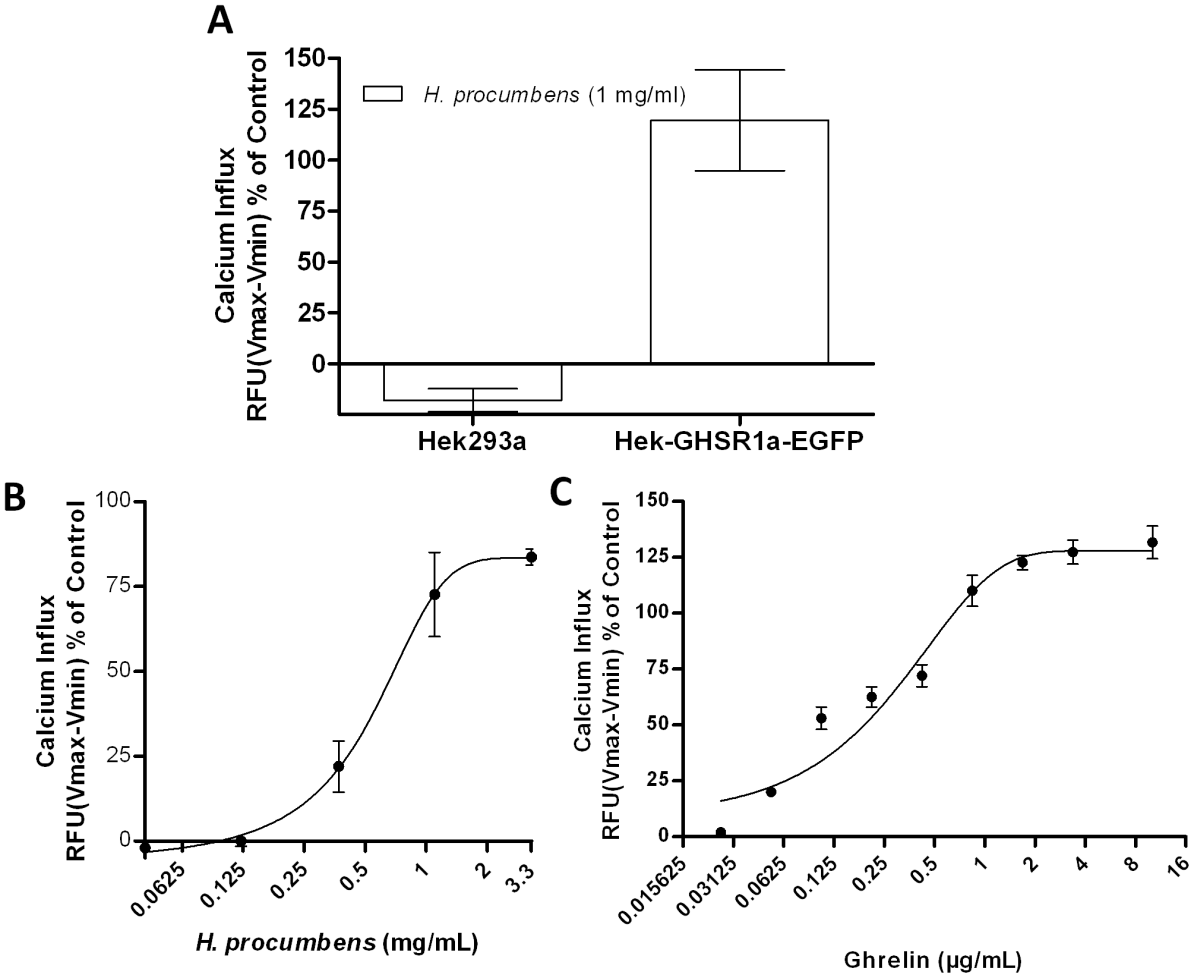
*H. procumbens* root extract induces GHS-R1a-mediated calcium influx. Calcium influx in Hek293a (wild type) cells versus Hek-GHS-R1a-EGFP cells (**A**) and dose response curves of *H. procumbens* root extract (**B**) and ghrelin (**C**) are depicted. Exposure to ghrelin, the endogenous ghrelin receptor ligand, and *H. procumbens* root extract potently increase intracellular calcium through activation of the GHS-R1a receptor in a dose dependent manner. Calcium increase was depicted as a percentage of maximal calcium increase as elicited by control in each separate experiment (3.33% FBS). The data represents the mean ± SEM of a representative experiment out of three independent experiments with each concentration point performed in triplicate.

GHS-R1a receptor modulation following *H. procumbens* root extract exposure was analysed in the calcium mobilization assay in Hek-GHS-R1a-EGFP cells and compared to the intracellular calcium increase mediated by the endogenous ligand, ghrelin (**Figure 2**). No calcium influx was observed in wild-type Hek cells (Hek293A wt) not expressing the GHS-R1a receptor when exposed to *H. procumbens* root extract (**Figure 2A**). In contrast, exposure of Hek cells stably expressing the GHS-R1a receptor to *H. procumbens* root extract did demonstrate a GHS-R1a receptor-mediated calcium influx in a dose dependent manner (**Figure 2B**). Efficacy (Emax) and the half maximal effective concentration (EC50) of *H. procumbens* mediated GHS-R1a receptor activation was compared to that of ghrelin (**Figure 2C**). Both ghrelin and the *H. procumbens* extract showed an efficacy >80% compared to control, indicating that both behave as full GHS-R1a receptor agonist, in the calcium mobilization assay. However, the EC50 was shown to be approximately 1000-fold lower (**table 2**) for the *H. procumbens* extract compared to ghrelin, which may not be surprising since the *H. procumbens* extract consists of a mixture of different compounds, resulting in an overall dilution of bioactive potency in its ability to activate the GHS-R1a receptor. Competing Interest statement, activation of the GHS-R1a receptor by its endogenous agonist ghrelin was shown to result in an increased intracellular calcium influx above 100% at concentrations exceeding 1 µg/mL (**Figure 2C**). This may be due to the additive effect of several mechanisms of calcium mobilization, including IP3 release of intracellular stores from the endoplasmic reticulum, entry of calcium across the plasma membrane via calcium permeable channels, and by mechanisms that export or re-sequester calcium after receptor activation. This warrants further investigations.

**Table 2:** Efficacy and EC50 of *H. procumbens* extract for GHS-R1a receptor-mediated calcium mobilization.

	Ghrelin	*H. procumbens* extract
**Emax (%)**	121.8±9.8	87.3±14.7
**EC50 (mg/mL)**	0.215*10^−3^±0.06	0.351±0.11

Emax and EC50 values were obtained using GraphPad Prism software.

**Figure 3:**
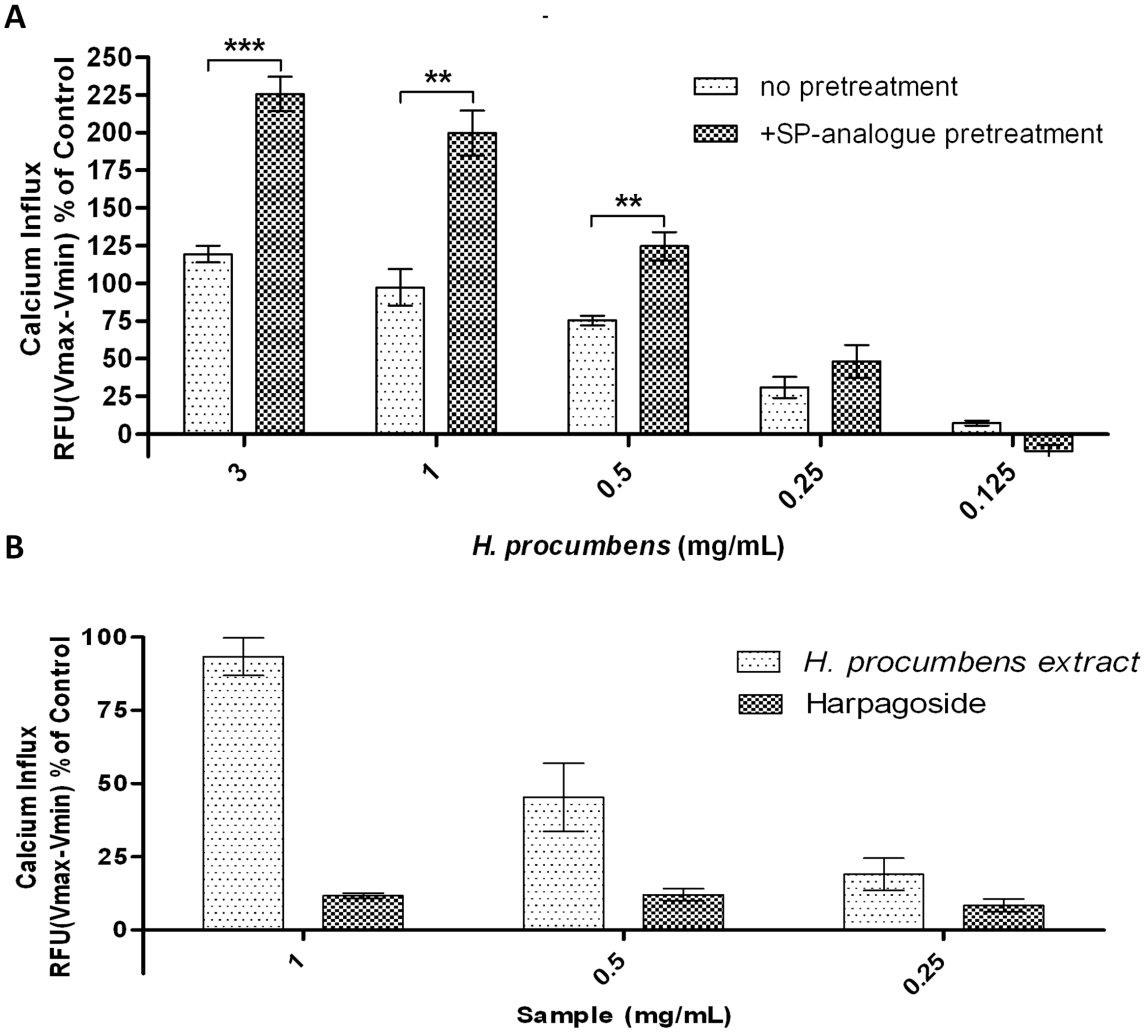
*H. procumbens* root extract specifically activates GHS-R1a receptor independent of harpagoside. Calcium mobilization upon exposure to *H. procumbens* root extract and harpagoside in Hek cells stably expressing the GHS-R1a receptor as an EGFP fusion protein. *H. procumbens* root extract induced GHS-R1a receptor activation was enhancedfollowing attenuation of constitutive receptor activity by pre-treatment with the GHS-R1a receptor inverse agonist, [D-Arg1, D-Phe5, D-Trp7,9, Leu11]-substance P (500 nM, SP-analogue) (**A**). The iridoid glycoside harpagoside, one of the main compounds present in *H. procumbens*, did not show an enhanced GHS-R1a receptor-mediated calcium influx, suggesting that the activity observed in this extract is due to others compounds present (**B**). Graph represents the mean ± SEM of a representative experiment from three (**A**) or two (**B**) independents experiments with each concentration point performed in triplicate. Intracellular calcium increase was depicted as a percentage of maximal calcium increase as elicited by control (3.3% FBS). ***p<0.001, **p<0.01 compared with no [D-Arg1, D-Phe5, D-Trp7,9, Leu11]-substance P pre-treatment.

In a second experiment, the *H. procumbens* root extract-mediated calcium mobilization following pre-treatment with the GHS-R1a receptor specific inverse agonist peptide, [D-Arg1, D-Phe5, D-Trp7,9, Leu11]-substance P (SP), was analysed (**Figure 3A**). SP-analogue was reported as a potent inverse agonist for the GHS-R1a receptor attenuating its high ligand independent basal activity [37]. Consequently, exposure to SP-analogue has been shown to significantly increase membrane GHS-R1a receptor expression and sensitize receptor signalling [38], [39]. Calcium increase was not significant different following exposure to *H. procumbens* root extract concentrations at the lowest concentrations (0.25 and 0.125 mg/mL) of SP-analogue pre-treatment. However, *H. procumbens* root extract exposure following SP-analogue pre-treatment did significantly enhance the GHS-R1a receptor mediated calcium influx at 3, 1 and 0.5 mg/mL. Statistical significance was determined at t(4) = 8.409; *p*<0.001, t(4) = 5.314; *p*<0.01 and t(4) = 1.348; *p*<0.01, respectively.

Next, we analysed harpagoside, the most studied compound in *H. procumbens*, for its ability to activate the GHS-R1a receptor *in vitro*. No increase in the intracellular calcium influx was observed after its exposure at different concentrations (**Figure 3B**). These results suggest that the *H. procumbens* root extract-mediated calcium mobilization is not due to the presence of harpagoside in the extract.

### GHS-R1a receptor internalization is not affected by *Harpagophytum procumbens* root extract

The GHS-R1a receptor has a high constitutive activity in the absence of ligand. Following ligand-mediated receptor activation a desensitization process occurs in order to protect the cell against receptor overstimulation [40]. This process of desensitization is a consequence of a combination of the uncoupling of the receptor from heterotrimeric G proteins and its internalization from membrane to intracellular compartments into endosomes [40]. Then, the receptor is marked for degradation or recycling back to the membrane and is a hallmark of receptor activation [41].

**Figure 4:**
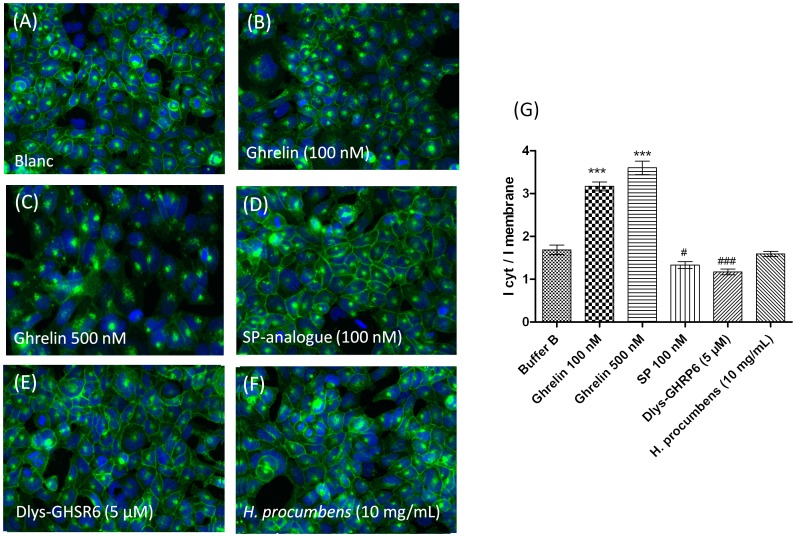
*H. procumbens* root extract does not internalize the GHS-R1a receptor. Hek cells stably expressing the GHS-R1a receptor as a C-terminal EGFP fusion protein were visualized using the IN Cell Analyser 1000 (GE Healthcare) following different treatments: untreated (**A**), ghrelin (**B**,**C**), [D-Arg1, D-Phe5, D-Trp7,9, Leu11]-substance P (SP-analogue) (**D**), (Dlys^3^)-GHRP-6 (**E**) and *H. procumbens* root extract (**F**) at the indicated concentrations for 1 h at 37°C. Ligand-mediated GHS-R1a-EGFP translocation is quantified following the EGFP fluorescent trafficking away from membrane into vesicles within the cytosol. Graph represents the mean ± SEM of the fluorescence intensity of perinuclear receptor expression versus plasma membrane receptor expression from a representative experiment out of two independent experiments with each treatment performed in triplicate (**G**). Significant increased internalization is depicted as *** *p*<0.001, and significant decreased internalization is depicted as ##*p*<0.01, #*p*<0.05 with respect to internalization obtained from assay buffer (blanc).

Internalization of the GHS-R1a receptor was investigated in Hek cells stably expressing the receptor as an EGFP-tagged fusion construct. GHS-R1a receptor trafficking could be monitored following analysis of EGFP fluorescent translocation from the cellular membrane into endosomes within the cytosol (**Figure 4**). Clear internalization of the GHS-R1a receptor could be observed after treatment with the endogenous agonist ghrelin at 100 and 500 nM (**Figure 4B, 4C**). Ghrelin-mediated GHS-R1a receptor internalization resulted in a high significant increased cytosol/membrane EGFP fluorescent intensity ratio (*p*<0.001) with respect to untreated cells (cells in assay buffer) (**Figure 4G**). In contrast, treatment with 100nM of the inverse agonist SP-analogue resulted in a higher GHS-R1a-EGFP expression in the membrane with respect to untreated cells (**Figure 4D**) and consequently showed a significant decreased cytosol/membrane EGFP fluorescent intensity ratio (*p*<0.05) (**Figure 4G**). In addition, the GHSR-R1a internalization after exposure to 5 µM of the ghrelin-receptor antagonist (Dlys^3^)-GHRP-6 was also analysed (**Figure 4E**). This antagonist has been widely used in *in vivo* and *in vitro* studies to antagonize the GHS-R1a receptor [34], [42]. Indeed, the GHS-R1a receptor antagonist, (Dlys^3^)-GHRP-6 significantly decreases the membrane/cytosol EGFP fluorescent intensity ratio (*p*<0.05) compared to untreated cells, which would correspond to higher levels of GHS-R1a receptor membrane expression (**Figure 4G**). Interestingly, the *H. procumbens* root extract did not significantly change GHS-R1a-EGFP fluorescence translocation, despite its high potency to induce a GHS-R1a receptor-mediated calcium influx (**Figure 4F, 4G**).

### *Harpagophytum procumbens* extract potently decreases cumulative food intake

**Figure 5:**
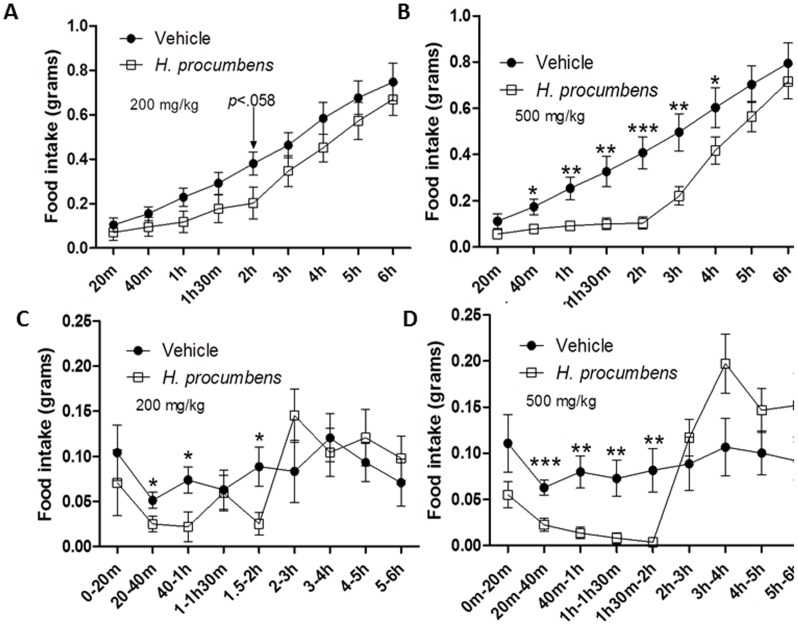
Anorexigenic effect of *H. procumbens* root extract in *ad libitum* conditions. Cumulative food intake (**A**,**B**) and food intake per time bin (**C,D**) in *ad libitum* fed C57BL/6 mice following intraperitoneal administration of *H. procumbens* root extract (200 and 500 mg/kg; 2.5% DMSO) and vehicle (saline; 2.5% DMSO). Results are depicted in line graphs ± SEM. Statistical significant differences compared to vehicle are depicted as * *p*<0.05, ** *p*<0.01 or *** *p*<0.001, n = 10 per group.

Finally, the effect of *H. procumbens* root extract on cumulative food intake was investigated in male C57BL/6 mice (n = 10 per cohort) during the light cycle (**Figure 5**). *H. procumbens* root extract (200 and 500 mg/kg in saline containing 2.5% DMSO) were administered to *ad libitum* fed mice via IP injection at 20 minutes before placement of food pellets in the cages (**Figure 5A, 5B**). Cumulative food intake was measured in regular intervals. An overall significant effect of exposure to 200 mg/kg *H. procumbens* root extract was observed F(1,18) = 1.761; *p*<0.201, as well as a significant main effect of time F(2.688,48.377) = 147.786; *p*<0.001, and an interaction of time and drug, F(2.688,48.377) = 1.312; *p*<0.281. Exposure to *H. procumbens* at 500 mg/kg showed a significant interaction of time and drug F(2.202,39.640) = 4.634;*p*<0.013, as well as a significant main effect of time F(2.202,39.640) = 137.707;*p*<0.001, and a significant main effect of drug; F(1,18) = 5.680;*p*<0.028. The highest dose administration of *H. procumbens* root extract (500 mg/kg) significantly decreased cumulative food intake compared to vehicle up to 4 h (**Figure 5B**), while exposure to the lower dose (200 mg/kg) did not reach significance (**Figure 5A**). Nevertheless, the lower dose of the extract did attenuate cumulative food intake which almost reached statistical significance at 2 h (*p*<0.058). In addition, cumulative food intake in individual time bins was also studied to provide information on food patterning. Exposure to the lower dose reached significance in the time bins from 20 min to 40 min, 40 min to 1 hr and 1.5 hr to 2 hr (**Figure 5C**) while exposure to the highest dose of *H. procumbens* root extract (500 mg/kg) significantly reduced food intake in time bins 20 min to 40 min, 40 min to 1 h, 1 h to 1 h30 min and 1.5 h to 2 h (**Figure 5D**). During the food intake study, no aberrant behaviour was observed in the animals.

**Figure 6:**
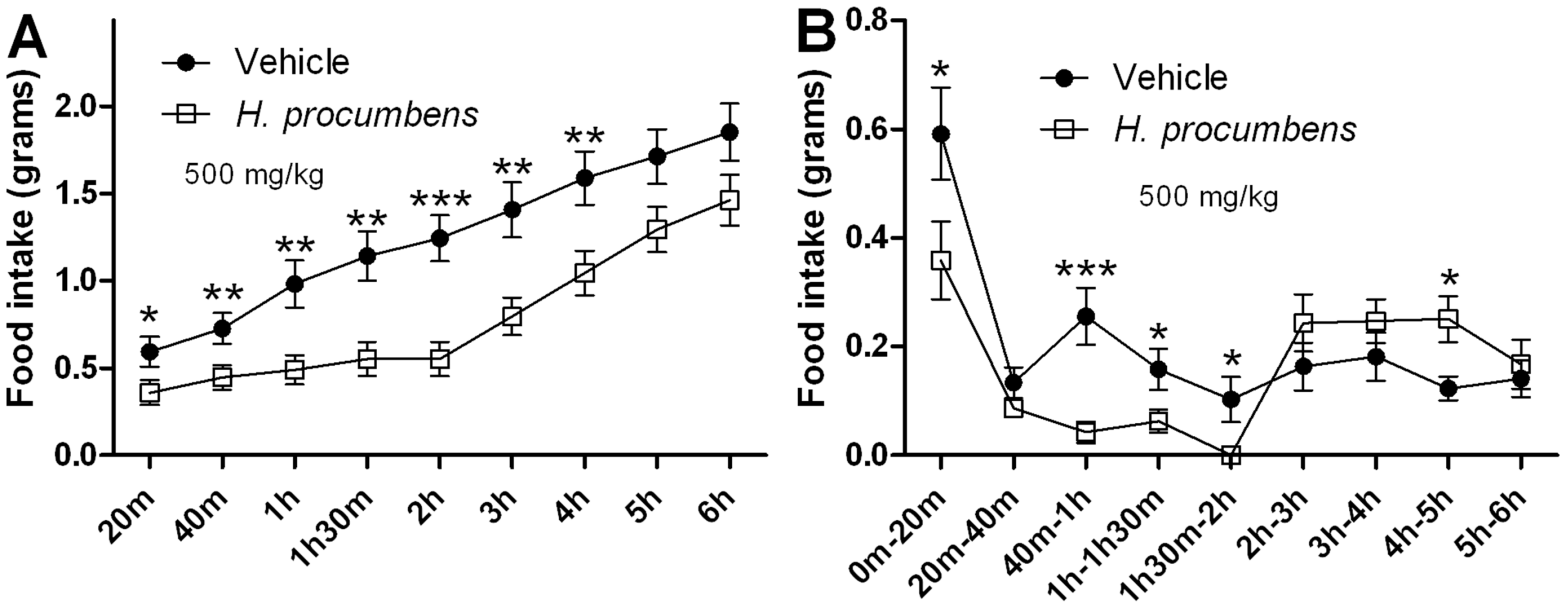
Anorexigenic effect of *H. procumbens* root extract in food restricted condition. Cumulative food intake (**A**) and food intake per time bin (**B**) in 16 h food restricted C57BL/6 mice following intraperitoneal administration of *H. procumbens* extract (500 mg/kg; 2.5% DMSO) and vehicle (saline; 2.5% DMSO). Results are depicted in line graphs ± SEM. Statistical significant differences compared to vehicle are depicted as * *p*<0.05, ** *p*<0.01 or *** *p*<0.001, n = 10 per group.

In addition, the anorexigenic effect of *H. procumbens* root extract in food-restricted mice was investigated (**Figure 6A, 6B**). A clear significant attenuation of cumulative food intake was shown following *H. procumbens* extract treatment (**Figure 6A**) with a significant interaction of time and drug; F(2.869,51.649) = 6.472;*p*<0.001 as well as a significant main effect of time; F(2.869,51.649) = 182.283;*p*<0.001 and a significant main effect of drug; F(1,18) = 8.330;*p*<0.01. A significant difference was observed between groups after 20 min, 40 min, 1 h, 1 h30 min, 2 h, 3 h and 4 h in cumulated food intake of *p*<0.05, *p*<0.01, *p*<0.01, *p*<0.01, *p*<0.001, *p*<0.01 and *p*<0.01 respectively. Again when analysing individual time bins significance was mainly observed in the time bins up to and including 2 h following food placement (**Figure 6B**), which normalized thereafter.

## Discussion

Traditionally, *H. procumbens* has been used as an herbal medicine for a variety of conditions, and currently it is mainly used as an anti-inflammatory agent and as an analgesic [21]. In addition, it has been traditionally (but anecdotally) used as a modulator of appetite [43]. However, scientific studies evaluating the effect on food intake are lacking. In this paper we show, to our knowledge for the first time, the ability of *H. procumbens* root extract to modulate the GHS-R1a receptor, which is a key receptor implicated in appetite stimulation following activation by its agonist ghrelin. The *H. procumbens* extract was able to potently stimulate an intracellular calcium influx *in vitro*. In addition, the *H. procumbens* root extract-mediated GHS-R1a receptor modulation was enhanced in response to GHS-R1a receptor sensitization following pre-treatment with the inverse agonist peptide, [D-Arg1, D-Phe5, D-Trp7,9, Leu11]-substance P (SP). It was shown that the *H. procumbens* root extract-mediated GHS-R1a receptor response is not due to the presence of the main iridoid glycoside harpagoside, contained within the plant, suggesting that other compounds in the extract are responsible for the interaction with the GHS-R1a receptor.

We also analysed the effect of *H. procumbens* extract on GHS-R1a receptor internalization into endosomes, which is a characteristic of full receptor activation and subsequently follows receptor desensitization [41]. Both desensitization and internalization processes provide essential physiological “feedback” mechanisms that protect against both acute and chronic overstimulation of receptors [44], [45]. However, no internalization of the receptor was observed following *H. procumbens* root extract exposure suggesting that this extract does not act as a full GHS-R1a receptor agonist. Inverse agonists, like SP have been shown to decrease constitutive activity leading to enhanced ligand-mediated calcium signalling [38], [39], which is confirmed in this study as *H. procumbens*-mediated calcium increase through the GHS-R1a receptor is increased following pre-treatment with SP. However, *H. procumbens* root extract did not increase GHS-R1a receptor expression on the membrane like SP did and it is thus unlikely to act in a similar matter as an inverse agonist.

Receptors are phosphorylated by G-protein coupled receptor kinases (GRK) following agonist-mediated activation and this process activates proteins involved in G protein-coupled receptors (GPCR) internalization [44], [45], such as β-arrestin, which is the most widely standard adaptor for GPCR endocytosis [46]. Indeed, GHSR-1a receptor stimulation by the endogenous ligand ghrelin induces β-arrestin recruitment and activates the mitogen-activated protein kinase (MAPK) pathway (for review see [47], [48]). Perhaps, β-arrestin recruitment is not mediated by *H. procumbens*-mediated GHS-R1a receptor activation, as β-arrestin independent recruitment has also been demonstrated, which warrants further investigations. Thus, we show that the *H. procumbens*-mediated intracellular calcium signalling alone is not sufficient to promote GHS-R1a receptor internalization and full receptor activation. Interestingly, a significant dose-dependent decrease in food intake was observed following intraperitoneal administration of *H. procumbens* root extract in *ad libitum* fed mice as well as a decrease in intake in food restricted mice. The major significant appetite effect of *H. procumbens* occurs within the first 2 hours of administration (**Figure 5** **,** **6**). This early effect on appetite is in line with the orexigenic effects of the endogenous GHS-R1a receptor ligand, ghrelin, which are also only observed within the first 2 hours of administration [34].

We hypothesize that the *H. procumbens* root extract interacts with the GHS-R1a receptor, as elicited by increased intracellular calcium influx, but does not lead to subsequent GHS-R1a receptor internalization and, therefore, does not act as full GHS-R1a receptor agonist. This may suggest that the GHS-R1a receptor internalization is required for further down-stream orexigenic effect *in vivo.* It is indeed tempting to speculate that the orexigenic effects of the endogenous ligand ghrelin are dependent on GHS-R1a receptor internalization, which occurs immediately following calcium signalling. Moreover, we suggest that modulation of the GHS-R1a receptor by the *H. procumbens* extract may decrease the availability of the receptor to the orexigenic effects of ghrelin, which may explain the observed anorexigenic effects.

Further investigations are needed to clarify the precise GHS-R1a receptor-mediated intracellular signal transduction pathways and correlate these to physiological behaviours. A recent study has shown that GHS-R1a receptor knock-down, inverse agonism, or desensitization can exert the same biological effect under certain circumstances [49]. However, in relation to food intake, GHS-R1a receptor inverse agonism has been reported to reduce appetite and body weight gain [49] in contrast to the orexigenic effects of ghrelin. Interestingly, biased agonism has also been reported for GPCRs modulation leading to different active receptor conformations adding to the complexity of GHS-R1a receptor signalling (for review [48]). Hence, different intracellular signal transduction pathways may be triggered by ghrelin, inverse agonists and *H. procumbens* extract.

Furthermore, we cannot rule out that the *H. procumbens* root extract interacts with other receptors implicated in satiety, such as the serotonin receptors (5-HT_1B_, 5-HT_2C_ and 5-HT_6_), cholecystokinin receptor (CKK-A) and glucagon-like peptide-1 receptor (GLP-1R), suppressing appetite. However, to our knowledge no such interactions have been reported to date. Future studies using GHS-R1a receptor antagonists or GHS-R1a knock-out mice may be able to further delineate the molecular mechanism of the GHS-R1a receptor-dependent anorexigenic effects of *H. procumbens*. Finally, it would also be interesting to investigate if the *H. procumbens*-mediated anorexigenic effect is maintained following oral administration and if the bioactive has proteolytic stability in transit.

Following compositional analysis, the most abundant compounds in the dried *H. procumbens* root powder were demonstrated as carbohydrates. Among these compounds, the most potential active constituent of the extract are iridoids glycosides (for review see [20]). In this study we show that harpagoside, previously demonstrated as the main iroid glycoside in *H. procumbens* [50], does not have any GHS-R1a activating potential. Therefore, harpagoside is not implicated in the interaction between *H. procumbens* extract and the GHS-R1a receptor. However, others iridoids glycosides may be implicated. In addition, the dried *H. procumbens* root was also rich in fibre. Several studies have shown that consumption of diets rich in fibre lead to beneficial anti-obesity effects such as increased satiety, reduced hunger, reduced food intake, and body weight loss (for review see [51]). Fibre exerts these anti-obesity effects by acting in the gastrointestinal tract through different mechanisms such as increasing gastric distension, delaying gastric emptying, digestion and absorption of nutrients, increasing insulin and glycemic responses, affecting gut hormones secretion such as GLP-1, peptide YY and neurotensin, reducing the absorption of fat and increasing the fecal energy excretion (for review see [51]). Therefore, fibre may also be potentially implicated in the decreased food intake observed *in vivo* by affecting gastrointestinal digestion process. However, further analyses are needed to investigate a possible interaction of fibre with the ghrelin receptor, which may be also implicated in the anorexigenic effect of *H. procumbens*. Future studies are needed to identify the specific bioactive responsible for the appetite suppressant effects of *H. procumbens.* Nevertheless, as demonstrated in this study, the crude *H. procumbens* extract has potent anorexigenic effects, which would be sufficient to be utilized as a natural anti-obesity treatment in its un-purified form. This significantly contributes to its potential commercial application.

We conclude that *H. procumbens* root extract is a novel source for potent anti-obesity bioactives with GHS-R1a mediated appetite suppressant effects. Therefore, *H. procumbens* root extract may represent a possible natural alternative which may be safer and more attractive compared to current pharmacological drugs, which are often associated with several side effects. Hence, the identification of the GHS-R1a receptor modulating bioactive from *H. procumbens* is poised to have important therapeutic potential in obesity and obesity related diseases.

## Supporting Information

Text S1 Compositional analysis of the unprocessed dried *Harpagophytum procumbens* root powder. **Text S1A.** Ash. **Text S1B.** Moisture**. Text S1C.** Lipids. **Text S1D.** Saccharides**. Text S1E.** Total fibre. **Text S1F.** Protein**. Text S1G.** Polyphenols.
(DOC)
