# Supplementary material for: Devil's Claw to Suppress Appetite—Ghrelin Receptor Modulation Potential of a Harpagophytum procumbens Root Extract
Source: PLoS One. 2014 Jul 28;9(7):e103118. doi: 10.1371/journal.pone.0103118 (PMC4113378; doi:10.1371/journal.pone.0103118)
Supplement: Text S1 — Compositional analysis of the unprocessed dried Harpagophytum procumbens root powder. Text S1A. Ash. Text S1B. Moisture. Text S1C. Lipids. Text S1D. Saccharides. Text S1E. Total fibre. Text S1F. Protein. Text S1G. Polyphenols. (DOC) [file pone.0103118.s001.doc]

**Text S1 Compositional analysis of the unprocessed dried *Harpagophytum procumbens* root powder**

All chemical composition analyses were done in duplicate from the dried *H. procumbens* root powder.

**Text S1A Ash**

Ash content of the sample was analysed using the Official Method 942.05 from the Association of Analytical Communities (AOAC) international [22]. Briefly, 200 mg of sample was weighed into a porcelain crucible and placed in a temperature controlled furnace at 600 °C for 2 h. Then, the crucible was cooled in a desiccator and weighed immediately. Ash content was reported as mass percentage.

**Text S1B Moisture**

Moisture content was analysed according to the AOAC Official Method 934.01 [22]. Briefly, 100 mg of sample was dried to constant weight at 100 °C under pressure of <100 mm Hg. Moisture was reported as percentage of loss in weight.

**Text S1C Lipids**

Lipid content was analysed according to the reference method 659:2009 from the International Organization for Standardization (ISO) [23], which specifies the determination of the hexane extract of oilseeds. To this end, 2 g of sample was extracted with 200 mL hexane in a Soxhlet system (Fisher Scientific, Madrid, Spain) for 9 h. The total lipid content was expressed as mass percentage.

**Text S1D Saccharides**

Polysaccharides and related substances were analysed by a colorimetric method according to Dubois *et al*. [24]. Simple sugars, oligosaccharides, polysaccharides, and their derivatives, including the methyl ethers with free or potentially free reducing groups undergo a colorchange from orange to a stable yellow colour, following treatment with phenol and concentrated sulfuric acid. To determine saccharides content, 0.5 g of sample was extracted with 200 mL of 95% ethanol in a Sowhlet system for 16 h. Then, the ethanol sample extract was filtered and the volume was adjusted to 200 mL. Next, 100 µL extract aliquots was dissolved in 1 mL of distilled water and 1 mL of 5% phenol and 5 mL of concentrated sulphuric acid was added. The mixture was shaken for 10 min at room temperature. Afterward, the mixture was incubated for 15 min at 30 °C. Finally, the absorbance was measured at 480 nm. Final saccharides content was determined using a standard curve of glucose as a reference. Blanks were prepared by substituting the sample with distilled water.

**Text S1E Total fibre**

The determination of total, soluble and insoluble fibre was carried out by an enzymatic-gravimetric method according to Lee, Prosky and Devries [25]. Briefly, 1 g sample was dissolved in 40 mL of 0.05 M ethanesulfonic acid (MES)/tris(hydroxymethyl)aminomethane (TRIS) buffer (Sigma-Aldrich), pH 8.2. Then, 50 μL of a heat-stable α-amylase solution (A3306-10mL, Sigma-Aldrich) was added and the mixture was incubated for 15 min at 95-10 °C. Afterward, 100 μL of 50 mg/mL alcalase solution prepared in MES/TRIS buffer was added and the solution was incubated under shaking at 60 ºC for 30 min. Next, the pH was adjusted to 4.5 and 300 μL of an amyloglucosidase solution (A9913-10mL, Sigma Aldrich) was added. The mixture was incubated under shaking at 60 °C for 30 min. Then, 225 mL of 96% ethanol was added and the mixture was incubated for 1 h at room temperature. Afterward, the sample was filtered by 0.45 μm filters and the precipitate was washed sequentially with 78% ethanol, 96% ethanol and acetone. Finally, the precipitate was dried at 110 ºC. Protein and ash content were analysed according the corresponding methods described in this section (S1.1 and S1.6). Fibre content was calculated as follow:

Fibre (%) = Precipitate (g) – Protein (g) – Ash (g) / Sample (g) x 100

**Text S1F Protein**

Peptides and proteins were quantified by amino acid analysis according to Hidalgo, Alaiz and Zamora [26]. Briefly, 10 mg of sample was hydrolysed in presence of D, L-α-aminobutyric acid as internal standard with 1 mL of 6.0 M hydrochloric acid for 20 h at 110 °C. The hydrolyzed sample was taken to dryness, dissolved in 3 mL of 1 M sodium borate buffer (pH 9.0), and derivatized with diethyl ethoxymethylenemalonate. Finally, amino acids were fractionated by reverse-phase high-performance liquid chromatography (HPLC) (Beckman-Coulter, Brea, CA, USA) with UV detection at 280 nm using a previously described gradient [46]. Protein content was calculated from amino acid data.

**Text S1G Polyphenols**

Polyphenols content was determined using the Folin-Ciocalteou reagent (Sigma-Aldrich) as described by Singleton, Orthofer and Lamuela-Raventos [27]. This reagent is a mixture of [phosphomolybdate](http://en.wikipedia.org/wiki/Phosphomolybdate) and [phosphotungstate](http://en.wikipedia.org/wiki/Phosphotungstate) that react with phenolic and [polyphenolic](http://en.wikipedia.org/wiki/Polyphenol) compounds to form chromogens which can be detected spectrophotometrically. Briefly, 1 mL of sample ethanol extract, obtained as described in S1.4., was diluted in 60 mL of distilled water. Next, 5 mL of Folin-Ciocalteou reagent was added and the solution was mixed for 1-8 min. Then, 15 mL of 20% sodium carbonate solution was added and the volume was adjusted to 100 mL. After 2 h, absorbance was measured at 765 nm and polyphenols content was determined using a standard curve of catechin.
